# Epidermolysis Bullosa with Hypertrophic Pyloric Stenosis in a Newborn

**Published:** 2015-10-01

**Authors:** Mahdi Ben Dhaou, Saloua Ammar, Hamdi Louati, Hayet Zitouni, Mohamed Jallouli, Riadh Mhiri

**Affiliations:** Department of Pediatric Surgery, Hedi Chaker Hospital, Sfax, Tunisia

**Keywords:** Epidermolysis Bullosa, Hypertrophic pyloric stenosis, Newborn

## Abstract

Epidermolysis bullosa (EB) is an inherited blistering disorder characterized by the fragility of the skin and mucous membranes. Extracutaneous manifestations can be associated. We report a unique concomitant occurrence of EB and hypertrophic pyloric stenosis in a newborn.

## INTRODUCTION

Epidermolysis bullosa is heterogeneous group of skin fragility syndromes with the diagnosis hallmark of blistering and erosions of the skin. In addition to skin involvement, different forms of EB can be associated with extracutaneous manifestations. These include hair, nail and tooth abnormalities, ocular findings, and fragility of the epithelia in upper respiratory, urogenital and gastro-intestinal tracts [1-3]. Particularly interesting are two forms of EB, one associated with late onset muscular dystrophy and another one with congenital pyloric atresia. Herein we present an unusual association of EB with hypertrophic pyloric stenosis (HPS) in a new born.

## CASE REPORT

A female infant presented with (family history of death by epidermolysis bullosa) recurrent non bilious vomiting. Antenatal scan showed distended stomach at the end of pregnancy. Physical examination showed a soft abdomen. Abdominal radiography showed a distended stomach. Pyloric atresia suspected and surgical exploration was done. There was no atresia; pyloric olive was hypertrophied. An extra-mucosal pyloromyotomy was performed (Fig.1). The baby tolerated feeds after operation. At the age of six days, multiple bullae and erosive patches were developed over the entire body. Biopsy taken from bullae confirmed EB. The patient died from a septic shock secondary to superinfection of skin lesions at the 20th day of her life.

**Figure:**
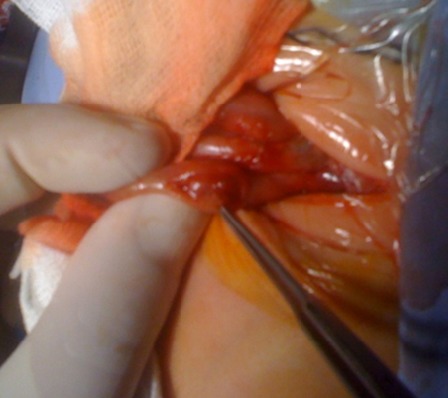
Figure 1: HPS

## DISCUSSION

EB has been divided into three major categories: EB simplex, junctional EB, and dystrophic EB. EB with pyloric atresia (EBPA) is rare and classified as junctional EB [4]. Complete obstruction by a fibrotic membrane can be explained by narrowing of the pylorus and proximal part of duodenum associated with the gastric submucosal connective tissue. These findings are frequently associated with inflammation [4]. In our case during surgical exploration, there was no inflammatory lesion, pyloric and duodenum lumens were patent. There was no mucosal membrane (no web, no diaphragm). Only pyloric muscle was hypertrophied. We found another case of pyloric stenosis associated with dominant dystrophic epidermolysis bullosa on literature search [1]. The association between HPS and EB may be coincidental. Our case might develop HPS at the first week of life and association with EB may be coincidental.

The general prognosis of EB is poor because of systemic manifestations such as electrolytes imbalance, failure to thrive, protein-losing enteropathy and septicemia [4]. In some cases, skin fragility is so severe that the affected children die from complications within a few days postpartum [3], as happened in our case.

## Footnotes

**Source of Support:** Nil

**Conflict of Interest:** Nil
